# Use of cortical hemodynamic responses in digital therapeutics for upper limb rehabilitation in patients with stroke

**DOI:** 10.1186/s12984-024-01404-y

**Published:** 2024-07-10

**Authors:** Jinuk Kim, Eunmi Kim, Su-Hyun Lee, Gihyoun Lee, Yun-Hee Kim

**Affiliations:** 1 Center for Neuroscience Imaging Research, Institute for Basic Science (IBS), Suwon, 16419 Republic of Korea; 2 Department of Physical and Rehabilitation Medicine, Sungkyunkwan University School of Medicine, Suwon, 16419 Republic of Korea; 3 Department of Biomedical Engineering, Chonnam National University, Yeosu, 59626 Republic of Korea; 4 School of Healthcare and Biomedical Engineering, Chonnam National University, Yeosu, 59626 Republic of Korea; 5 Myongji Choonhey Rehabilitation Hospital, Seoul, 07378 Republic of Korea

**Keywords:** Stroke, Functional near-infrared spectroscopy, Cerebral hemodynamic response, Digital rehabilitation, Upper limb motor function

## Abstract

**Background:**

Stroke causes long-term disabilities, highlighting the need for innovative rehabilitation strategies for reducing residual impairments. This study explored the potential of functional near-infrared spectroscopy (fNIRS) for monitoring cortical activation during rehabilitation using digital therapeutics.

**Methods:**

This cross-sectional study included 18 patients with chronic stroke, of whom 13 were men. The mean age of the patients was 67.0 ± 7.1 years. Motor function was evaluated through various tests, including the Fugl–Meyer assessment for upper extremity (FMA-UE), grip and pinch strength test, and box and block test. All the patients completed the digital rehabilitation program (MotoCog^®^, Cybermedic Co., Ltd., Republic of Korea) while being monitored using fNIRS (NIRScout^®^, NIRx Inc., Germany). Statistical parametric mapping (SPM) was employed to analyze the cortical activation patterns from the fNIRS data. Furthermore, the K-nearest neighbor (K-NN) algorithm was used to analyze task performance and fNIRS data to classify the severity of motor impairment.

**Results:**

The participants showed diverse task performances in the digital rehabilitation program, demonstrating distinct patterns of cortical activation that correlated with different motor function levels. Significant activation was observed in the ipsilesional primary motor area (M1), primary somatosensory area (S1), and contralateral prefrontal cortex. The activation patterns varied according to the FMA-UE scores. Positive correlations were observed between the FMA-UE scores and SPM t-values in the ipsilesional M1, whereas negative correlations were observed in the ipsilesional S1, frontal lobe, and parietal lobe. The incorporation of cortical hemodynamic responses with task scores in a digital rehabilitation program substantially improves the accuracy of the K-NN algorithm in classifying upper limb functional levels in patients with stroke. The accuracy for tasks, such as the gas stove-operation task, increased from 44.4% using only task scores to 83.3% when these scores were combined with oxy-Hb t-values from the ipsilesional M1.

**Conclusions:**

The results advocated the development of tailored digital rehabilitation strategies by combining the behavioral and cerebral hemodynamic data of patients with stroke. This approach aligns with the evolving paradigm of personalized rehabilitation in stroke recovery, highlighting the need for further extensive research to optimize rehabilitation outcomes.

**Supplementary Information:**

The online version contains supplementary material available at 10.1186/s12984-024-01404-y.

## Background

Stroke, which has become increasingly prevalent with the global aging population, poses a significant public health challenge, leading to long-term disabilities and substantial burden on healthcare systems [1]. This condition results from cerebral vascular events that cause brain damage, which manifests in various impairments ranging from motor and cognitive to emotional and linguistic difficulties, substantially affecting individuals’ quality of life [2]. Effective rehabilitation is essential for improving functional recovery, reducing disabilities, and enabling the reintegration of stroke survivors into daily life, thus alleviating the socioeconomic burdens on their families and healthcare systems [3].

To create more engaging and interactive rehabilitation experiences, recent advancements in digital technology have introduced innovative approaches to rehabilitation, employing wearable technology, gamification principles, and virtual reality (VR) systems [4, 5]. These digital rehabilitation programs leverage advanced sensors and data analytics for precise patient assessment and personalized treatment plans [6]. Furthermore, artificial intelligence algorithms process data obtained through digital platforms, optimizing therapeutic outcomes by tailoring rehabilitation programs to individual needs and enhancing treatment effectiveness and personalization [7, 8].

Understanding neuroplasticity, which refers to the brain’s ability to create new neural connections, is fundamental for the development of effective neurorehabilitation strategies [9, 10]. This concept underpins treatments aimed at leveraging neural plasticity for recovery, supported by functional brain imaging research that highlights the brain’s capacity for reorganization and adaptation following a stroke [10]. Despite the recognized benefits of digital technologies in rehabilitation, the detailed relationship between cortical activation and rehabilitation outcomes remains unclear. Notwithstanding its limitations, functional near-infrared spectroscopy (fNIRS) offers advantages such as low cost, portability, and resilience to motion artifacts, making it a promising tool for understanding and using brain activation and plasticity in digital rehabilitation [11, 12].

This preliminary study was conducted to investigate cortical activation during stroke rehabilitation, as indicated by cerebral hemodynamic signals measured through fNIRS during digital rehabilitation. The first objective was to determine the characteristics of brain activation captured by cerebral hemodynamic response signals during digital rehabilitation. The second objective was to determine whether machine learning algorithms can effectively classify brain signals and elucidate patients’ functional status. The third objective was to improve our understanding of the intricate relationship between neurophysiological changes and functional motor performance in digital rehabilitation.

## Methods

### Participants

The study enrolled patients with chronic stroke aged 19–80 years who had experienced a stroke at least 6 months before enrolment. Eligible participants had unilateral subcortical lesions and mild-to-moderate upper extremity impairment, as indicated by Fugl–Meyer assessment for upper extremity (FMA-UE) scores of 25–57 [13, 14], and were capable of grasping and releasing objects. To ensure sufficient cognitive function for inclusion, a score of at least 24 in the Korean version of the Mini-Mental State Examination was required [15]. Patients with neurological disorders other than stroke, major psychiatric disorders (e.g., schizophrenia, bipolar disorder, dementia, or severe spatial/temporal neglect or apraxia), and musculoskeletal disorders or conditions preventing the safe use of medical devices (e.g., implantable electronic devices, cardiac diseases, pregnancy, skin diseases, open wounds, high fever, or infectious diseases) were excluded.

Our cross-sectional study aimed to observe differences in brain activation during upper limb motor tasks, focusing on cerebral activity patterns rather than statistically proving clinical efficacy. To determine the sample size, we referenced previous studies that analyzed brain activation in similar tasks and research on the Fugl–FMA-UE scores, which was a critical motor function indicator for our target stroke patient population. Specifically, a study by Kim, H.G. et al. (2022) [16], which used upper limb FMA scores and conducted a two-sided paired *t*-test, indicated a sample size of 20 participants to achieve an effect size of 1.308, a significance level (α) of 0.05, and a power (1-β) of 85%, calculated using G*Power version 3.1 [17]. Further supporting this, studies that employed near-infrared spectroscopy for regional brain activation analysis during upper limb motor tasks, including 1 with 20 stroke patients [18] and another with 20 healthy adults engaged in video game-based tasks, also yielded satisfactory results with 20 participants [19]. Based on these precedents, our study set out to recruit a total of 20 participants.

The final analysis included 18 (13 men; mean age, 67.0 ± 7.1 years) of the 21 participants who initially consented after 1 withdrew consent and 2 were excluded due to missing data (Supplementary Fig. 1). The participants’ demographics are summarized in Table 1.

**Table 1:** Demographic characteristics of the participants

Characteristics			Value (*n* = 18)
Age (years)			67.0 ± 7.1 (51, 80)
Sex (male/female)			13/5
Stroke type (infarction/hemorrhage)			11/7
Lesion side (left/right)			12/6
Duration (months)			68.4 ± 54.1 (14, 206)
FMA upper extremity (score)			45.9 ± 7.8 (32, 57)
Hand grip strength test (kg)			6.0 ± 6.6 (0, 22)
Grip and pinch strength test (kg)			2.3 ± 1.4 (0, 5.5)
Box and block test (ea)			30.6 ± 17.2 (0, 54)
Nine-hole pegboard test (sec)			76.8 ± 79.5 (0, 298)
JTHFT		Writing (s)	65.8 ± 30.7 (19.2, 120)
		Card turning (s)	31.8 ± 35.8 (1.6, 120)
		Picking up small objects (s)	46.5 ± 39.1 (9.3, 120)
		Feeding (s)	30.4 ± 34.2 (8.9, 120)
		Checkers (s)	22.2 ± 36.6 (3.2, 120)
		Lift light (s)	32.8 ± 39.1 (5.1, 120)
		Light heavy (s)	33.0 ± 40.9 (5.1, 120)
MMSE-K			29.1 ± 1.3 [26, 30]
MEP	rMT of the affected hemisphere (%)		58.8 ± 13.9 (35, 80)
	Amplitude of the affected hemisphere (mV)		0.33 ± 0.33 (0.05, 1.31)
	Latency of the affected hemisphere (ms)		25.3 ± 3.2 (19.9, 30.8)

Values are expressed as mean ± SD (min, max). FMA, Fugl–Meyer assessment; JTHFT, Jebsen–Taylor hand function test; MEP, motor evoked potential; MMSE-K, Korean version of the Mini-Mental State Examination; rMT, resting motor threshold; SD, standard deviation. In the JTHFT, uncompleted tasks were marked as 120 s. Two data points were missing for the MEP of the affected hemisphere

### Experimental protocol

**Fig. 1:**
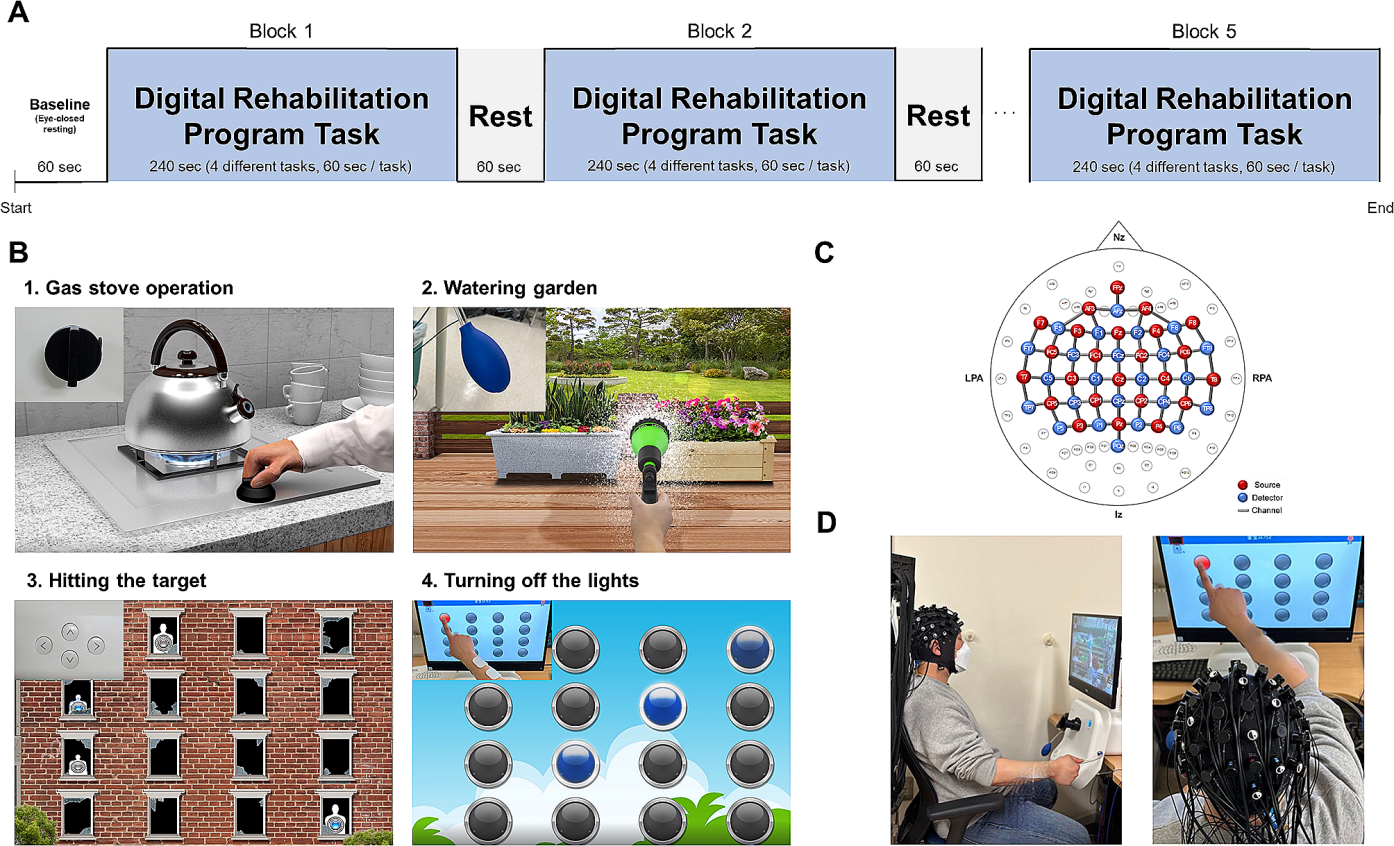
Study design. (**A**) Experimental paradigm. The paradigm consisted of four tasks in a box-car block design, with each task block lasting 4 min followed by a 1-min rest block. This sequence was repeated five times. The tasks varied and were randomized. (**B**) Digital rehabilitation program tasks. The upper-left corner of the figure shows the tools used in each task. (**C**) fNIRS topographical map. Red circles indicate 24 sources; blue circles, 24 detectors; and gray lines, 81 channels. (**D**) Experiment photo. A participant is performing the digital rehabilitation program and wearing an fNIRS device to measure their cerebral hemodynamic responses

This cross-sectional study was conducted to identify the characteristics of cerebral hemodynamic response during a digital rehabilitation program. The patients visited the hospital once to participate in the research. They underwent a series of upper limb motor function assessments before starting the digital rehabilitation program. These assessments included the FMA-UE, grip and pinch strength test, box and block test, nine-hole pegboard test (9HPT), Jebsen–Taylor hand function test (JTHFT), and motor evoked potential. All the patients performed the digital rehabilitation program for 25 min using their affected hand, during which their cerebral hemodynamic responses were monitored through fNIRS (Fig. 1D). The program started with a 60-s baseline in an eyes-closed resting state, followed by four tasks in a box-car block design. Each task block, lasting 4 min, comprised four individual tasks, each performed for 1 min. These tasks were randomized in order. Each task block was followed by a 1-min rest period and was repeated five times (Fig. 1A).

This study was conducted in accordance with the principles of the Declaration of Helsinki and approved by the Institutional Review Board of Samsung Medical Center, Republic of Korea (IRB No. SMC 2022-10-117).

### Multimodal digital rehabilitation system

This study used MotoCog® (Cybermedic Co., Ltd., Republic of Korea), a multimodal digital rehabilitation system designed to improve upper limb functions related to activities of daily living [20]. This system includes tools that mimic everyday items such as circular, O-shaped, and L-shaped handles, an air tube, a steering wheel, a gas range knob, and a touch screen. Each tool was equipped with sensors for motion detection and motor resistance assessment, facilitating haptic-based training in daily activities. The system encompasses 10 hand function training tasks. This study selected the following four tasks as representative of typical hand functions (Fig. 1B):

#### Gas stove operation

Participants hold a gas range knob and simulate turning on a gas stove as per the screen scenario through hand supination and pronation movements. The difficulty increases with the resistance and rotation angle.

#### Watering the garden

Participants hold an air bulb and mimic watering plants. It involves hand grasping and releasing motions. The force required to squeeze the bulb and the holding duration increase with increasing difficulty.

#### Hitting the target

Participants manipulate the touchpad on the device to align with the screen scenarios. Finger button-pressing actions are performed, with the number and speed of targets increasing as the difficulty intensifies.

#### Turning off the lights

Participants reach out and touch circles on the screen to turn off the lights. This task involves reaching and touching motions, and an increasing number of lights and additional untouchable colored lights appear at higher difficulties.

The participants’ task performance was recorded as scores. The tasks have adjustable difficulty levels (1–5). In this study, all tasks started at level 1 and progressed to level 5 based on the participant’s score. The difficulty adjustment criteria were as follows: decreased level in scores < 30, maintained level in scores 30–70, and increased level in scores > 70.

### fNIRS measurement

The regional cerebral hemodynamic response during the digital rehabilitation program was measured through fNIRS. In this study, a continuous-wave fNIRS measurement system (NIRScout®, NIRx Medical Technology, Berlin, Germany) on a platform compatible with multimodal inputs was utilized. The hemodynamic response signals were obtained as optical changes in a continuous waveform. The system utilized two wavelengths (760 and 850 nm), with a sampling rate of 10.25 Hz. The fNIRS optodes, including 24 LED light sources and 24 detectors, formed 81 source–detector channels. These channels were used to monitor cerebral hemodynamics across nearly the entire cortical area (Fig. 1C), including the frontal, motor, parietal, temporal, and occipital cortices. The precise locations of each channel can be confirmed through Supplementary Table 1. Montreal Neurologic Institute coordinates and Brodmann areas were determined by referencing information based on the existing 10/20 system for each channel [21]. The optodes were positioned according to the international 10/20 system with a channel distance (source to detector) of 3.0 cm. The cranial vertex (Cz), which was located beneath the first source, served as a marker for the consistent placement of the optodes. After locating the Cz position on the participant’s head, the fNIRS head cap was positioned accordingly.

The NIRStar 15.2 software (NIRx Medical Technologies) was used to capture signals, record raw fNIRS data, and obtain signal quality indicators for measurement channels following hardware calibration. The channels were evaluated for signal quality, and those with poor quality were excluded based on the following criteria: First, channels with a gain > 7 (inadequate light detection) were rejected. This gain was determined by the software during the calibration performed before each experiment. Gain values < 7, defined in the measurement software, corresponded to optical signals within the range of 0.09–1.4 V and noise levels < 2.5% [22]. In cases with poor signal quality during calibration, the contact between the scalp and the corresponding optodes was adjusted until the overall signal quality reached an acceptable level.

### Data processing and analysis of fNIRS

Cerebral hemodynamic responses were preprocessed and analyzed using the nirsLAB® software (v.2019.04; NIRx Medical Technologies). Discontinuities and spike artifacts in the raw signals from 81 channels were determined and replaced with the closest matching signals. The criterion for channel quality evaluation was based on a gain threshold of 8 and a coefficient of variation (CV) threshold of 7.5 [23]. In addition, instances of spikes and discontinuities were identified and then replaced with the nearest signals to enhance signal clarity [24]. To eliminate the baseline noise and filter out potential respiratory (about 0.3 Hz) and heart rate (about 1.0–1.6 Hz) signals, raw data were initially band-pass-filtered from 0.01 to 0.2 Hz [25]. Both oxyhemoglobin (oxy-Hb) and deoxyhemoglobin signals were captured; however, only the oxy-Hb concentration was included in the analysis owing to its higher signal-to-noise ratio [26]. The oxy-Hb concentration for each of the 81 channels was calculated from the preprocessed filtered data using the modified Beer–Lambert law [27].

Statistical parametric mapping (SPM) analysis was conducted to create a cortical activation map of the cerebral hemodynamic responses to oxy-Hb. A general linear model with a canonical hemodynamic response function was used in the SPM analysis to model the hypothesized oxy-Hb response and to test for significant cortical activation during the task block compared with the resting block [28]. Each task was executed for 1 min, with four distinct tasks consecutively executed in each block. To ensure statistical robustness and minimize potential carryover effects, the sequence of the blocks was repeated five times, with the order of the tasks within each block randomized in a balanced manner. In the SPM analysis, each 1-min task block was processed to separate and capture unique brain signals associated with each specific task through five repeated iterations. Subsequently, these signals were compared with the data from the resting blocks and then compared with the signals obtained during the 1-min resting blocks. Statistical analysis began with the calculation of individual-level beta-values from each participant’s fNIRS data, which were then used to derive oxy-Hb t-values for each participant, identifying specific cortical activation patterns within the participants. These individual oxy-Hb t-values were subsequently used in a group-level analysis to determine significantly activated channels (*P* < 0.05, not corrected) [29]. Furthermore, to determine regions with significant differences in oxy-Hb concentration, t-statistic maps derived from group analysis were plotted onto a conventional cortex template. All the participants were assumed to have lesions in the left hemisphere. Thus, the channels were flipped from left to right for patients with lesions in the right hemisphere. Individual-level t-values for all 81 channels were extracted, allowing for statistical analysis of the t-values for each channel.

### Statistical analysis

IBM SPSS Statistics for Windows version 20.0 (IBM Corp., Armonk, NY, USA) was used for statistical analysis. The Shapiro–Wilk test and Levene’s test were employed to evaluate data normality and the homogeneity of variance, respectively. Furthermore, Pearson’s correlation coefficients were utilized to explore the correlations among motor function scores, digital rehabilitation task scores, and oxy-Hb t-values from the fNIRS measurements. A *P*-value of 0.05 was used for all analyses.

In addition, the K-nearest neighbors (K-NN) algorithm was employed to classify FMA-UE severity in patients with stroke using the task performance scores and t-values in oxy-Hb as dependent variables, with FMA-UE severity as the predictor variable. The validity of the model was confirmed using a threefold cross-validation method, in which a sample was randomly divided into three subsets. One subset was used as a validation set in each validation cycle, and the remaining subsets were used to train the model. This cycle was repeated with each subset used as a validation set once. The average misclassification error, specificity, sensitivity, positive and negative predictive values, and overall accuracy were calculated to evaluate the model’s performance.

## Results

### Task performance results in the digital rehabilitation program

**Fig. 2:**
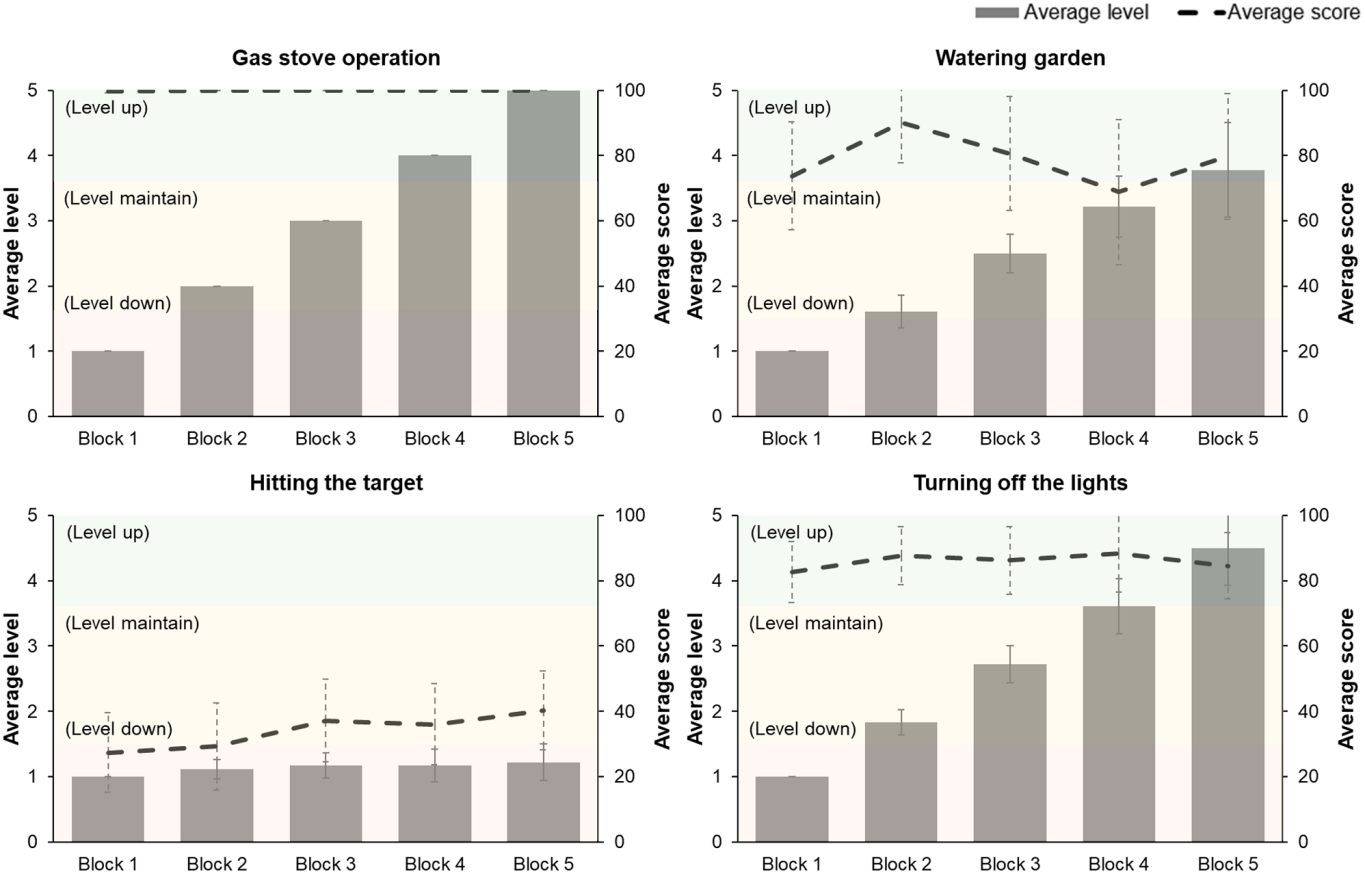
Average score and difficulty level of each task in the digital rehabilitation program. Each task started at level 1 difficulty and progressed up to level 5, with the difficulty increasing after each of the five repeated blocks based on the score. The difficulty level decreased if the score was < 30, remained the same if the score was 30–70, and increased if the score was > 70

The average performance scores and difficulty levels of each task in the digital rehabilitation program varied among the participants (Fig. 2). In the gas-stove-operation task, which required hand supination and pronation movements, all the participants reached the maximum difficulty level of 5, achieving an average score of 99.9 ± 0.2 (Supplementary Table 2). In the watering-the-garden task, which involved finger grasping and releasing motions, the average maximum difficulty level was 3.8 ± 1.4, with an average score of 78.6 ± 27.1. In the hitting-the-target task, which involved finger-tapping movements, the average maximum difficulty level was 1.2 ± 0.5, with an average score of 34.0 ± 20.9. Finally, in the turning-off-the-light task, the average maximum difficulty level was 4.5 ± 1.2, with an average score of 85.9 ± 18.1.

Table 2 shows the correlation between upper limb motor function and the average scores of each task in the digital rehabilitation program. The average correlation coefficient (r) was 0.21, which indicated no significant correlation with upper limb and hand function for the gas stove-operation task. Contrarily, the watering-the-garden task exhibited a strong significant correlation with all upper limb function assessment scores, except for 9HPT, which had an average correlation coefficient of 0.70 (*P* < 0.05). The hitting-the-target and turning-off-the-light tasks showed moderate statistical correlations with all upper limb function tasks (excluding 9HPT and the grip and pinch strength test), with average correlation coefficients of 0.54 (*P* < 0.05) and 0.61 (*P* < 0.05), respectively.

**Table 2:** Correlation between the upper limb motor function and the average score for each task

Characteristics		Gas stove operation	Watering the garden	Hitting the target	Turning off the lights
FMA upper extremity (score)		−0.36	**0.78** ^**^	**0.56** ^*^	**0.71** ^**^
Hand grip strength test (kg)		−0.25	**0.62** ^**^	**0.66** ^**^	**0.48** ^*^
Grip and pinch strength test (kg)		−0.24	**0.63** ^**^	0.37	0.37
Box and block test (ea)		−0.21	**0.83** ^**^	**0.60** ^**^	**0.75** ^**^
Nine-hole pegboard test (s)		0.17	−0.08	−0.21	−0.20
JTHFT	Writing (s)	0.31	**−0.66** ^**^	**−0.58** ^*^	**−0.69** ^**^
	Card turning (s)	0.17	**−0.83** ^**^	**−0.58** ^*^	**−0.59** ^**^
	Picking up small objects (s)	0.23	**−0.80** ^**^	**−0.62** ^**^	**−0.71** ^**^
	Feeding (s)	0.15	**−0.80** ^**^	**−0.49** ^*^	**−0.67** ^**^
	Checkers (s)	0.13	**−0.83** ^**^	**−0.50** ^*^	**−0.67** ^**^
	Lift light (s)	0.17	**−0.79** ^**^	**−0.62** ^**^	**−0.69** ^**^
	Light heavy (s)	0.17	**−0.79** ^**^	**−0.66** ^**^	**−0.81** ^**^

Values represent correlation coefficient (r), bold, and ^*^P < 0.05, ^**^P < 0.01. FMA, Fugl–Meyer assessment; JTHFT, Jebsen–Taylor hand function test; ea, each

### Cortical activation pattern during the digital rehabilitation program

**Fig. 3:**
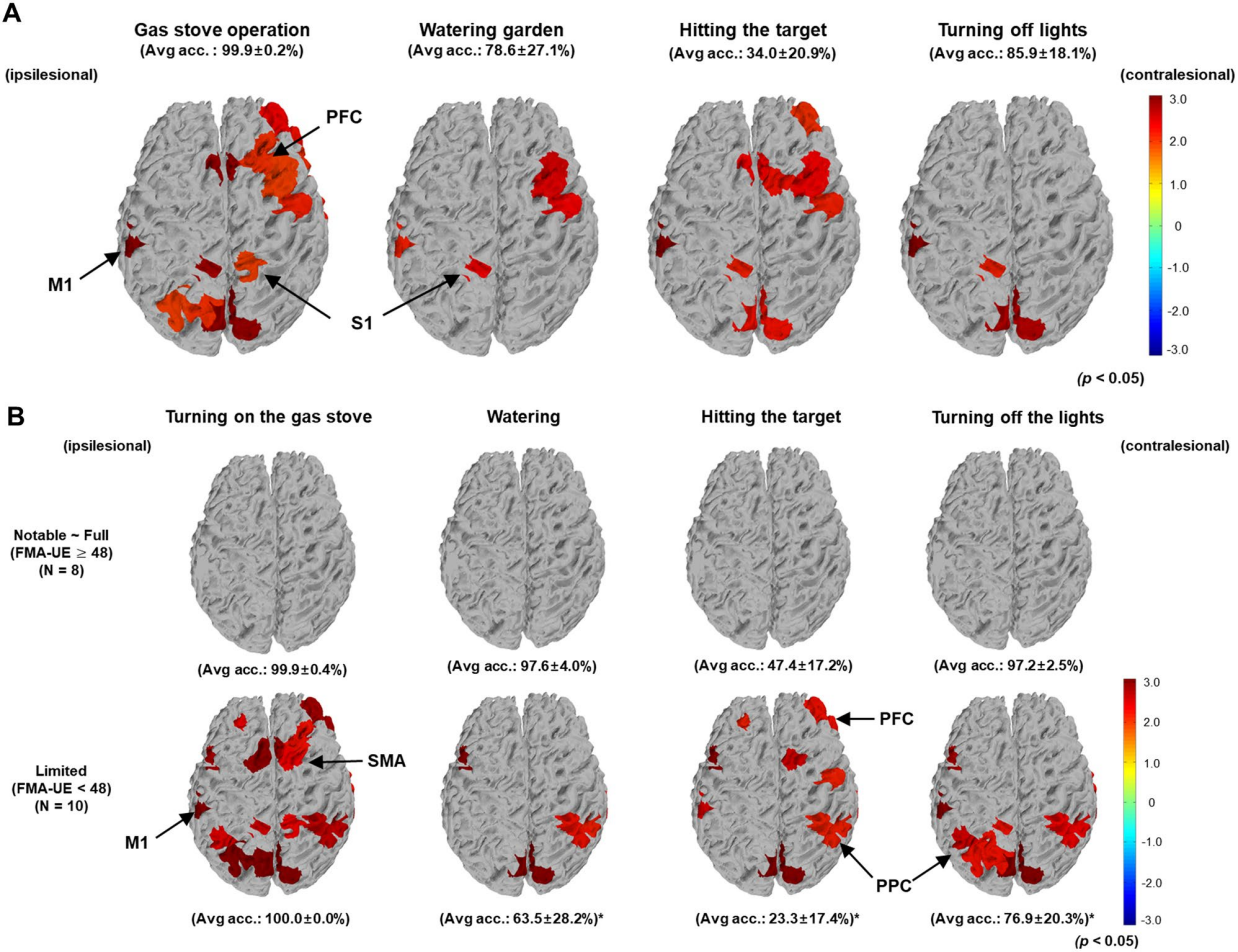
Average cortical activation patterns during the digital rehabilitation program. (**A**) Grand average cortical activation patterns for each task. The significant t-values from the statistical parametric mapping (SPM) analysis are represented for each channel (*P* < 0.05). (**B**) Subgroup analysis of cortical activation patterns based on the Fugl–Meyer assessment for upper extremity (FMA-UE) severity. M1, primary motor cortex; PFC, prefrontal cortex; PPC, posterior parietal cortex; SMA, supplementary motor area; S1, primary somatosensory cortex

Figure 3A illustrates the grand average cortical activation pattern obtained from 18 participants with chronic stroke executing tasks in the digital rehabilitation program compared with the resting state. The channels and colors represent the statistically significant t-values obtained from the SPM analysis. Significant activation was observed in the ipsilesional primary motor cortex (M1), primary somatosensory cortex (S1), and contralesional prefrontal cortex (PFC) in all tasks compared with the resting state.

A subgroup analysis was conducted using FMA-UE severity to explore hemodynamic brain activation characteristics based on functional levels in patients with stroke [30]. The analysis included 8 participants with notable to full upper limb function (scores of 48 points and above) and 10 participants with limited function (scores below 48 points). The group with notable to full upper limb function demonstrated no statistically significant cortical activation areas in the digital rehabilitation tasks (Fig. 3B). Conversely, increased use of the ipsilesional M1, supplementary motor areas (SMAs), bilateral posterior parietal cortices (PPC), and bilateral PFC was observed during the digital rehabilitation program in the group with limited function.

### Association between upper limb motor function, task performance, and cortical activation

Supplementary Fig. 2 shows the correlation map between FMA-UE scores, digital task performance scores, and oxy-Hb concentration t-values during the digital rehabilitation program. Red denotes a positive correlation; blue, a negative correlation; and bold markings, statistical significance.

**Fig. 4:**
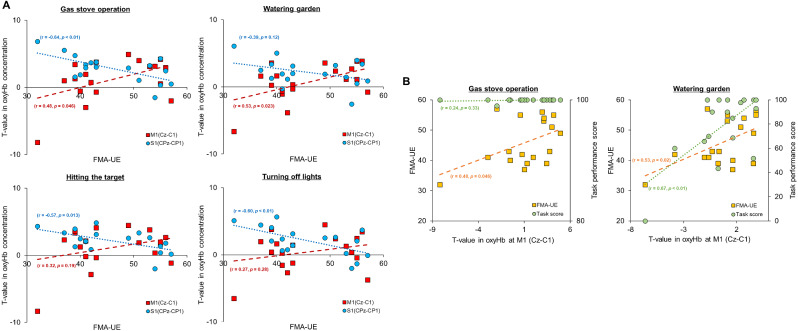
Association between upper limb motor function, task performance, and cortical activation. (**A**) Correlation between the FMA-UE scores and oxy-Hb t-values in ipsilesional M1 (Cz-C1 channel) and ipsilesional S1 (CPz-CP1 channel) in each task. (**B**) Correlation between the oxy-Hb t-values in ipsilesional M1 (Cz-C1 channel) with the FMA-UE scores and task performance scores during the task

Ipsilesional M1 generally exhibited a positive correlation across all tasks, whereas contralesional M1, SMA, S1, and PFC showed negative correlations in the relationship between FMA-UE and cortical activation. Notably, channels such as Cz-C1 consistently demonstrated statistically significant positive correlations in patients with left lesions, whereas channels such as F1-AF3, Fz-AFz, FC3-FC5, and CPz-CP1 showed significant negative correlations. In particular, a significant positive correlation was observed between the FMA-UE and the SPM t-values of oxy-Hb of the ipsilesional M1 (Cz-C1 channel) during the gas stove-operation and watering-the-garden tasks (Fig. 4A). However, the oxy-Hb t-values in ipsilesional S1 (CPz-CP1) showed a significant negative correlation with the FMA-UE scores in all tasks, except for the watering-the-garden task. The watering-the-garden task exhibited a trend toward a negative correlation, although not statistically significant (*P* = 0.12).

Contrarily, when analyzing the correlation between digital rehabilitation performance scores and cortical activation, tasks such as watering the garden, hitting the target, and turning off the lights, which showed moderate-to-strong correlations between function and scores as shown in Sect. 3.1, mirrored these outcomes in terms of correlation with upper limb function (Supplementary Fig. 2). However, the gas stove-operation task, which did not show correlation with function, exhibited a different pattern and did not show any significant correlation with cortical activation. In particular, the t-values of ipsilesional M1 demonstrated a significant correlation with the FMA-UE (*r* = 0.53, *P* = 0.02) and task performance scores (*r* = 0.67, *P* < 0.01) during the watering-the-garden task. Conversely, the t-values of ipsilesional M1 were only significantly correlated with FMA-UE (*r* = 0.48, *P* = 0.06) and demonstrated no correlation with task performance scores (*r* = 0.24, *P* = 0.33) in the gas stove-operation task (Fig. 4B).

### Classification of motor function using task scores and cortical activation

A K-NN classification analysis was conducted to determine whether cortical activation measured during the digital rehabilitation program facilitated the evaluation of participants’ functional levels. The predictor variable was FMA-UE severity, which was categorized as either “notable to full” or “limited.” The task scores and oxy-Hb t-values in the ipsilesional M1 (Cz-C1 channel) were used as dependent variables. This study compared the model performance when using only task scores versus both task scores and t-values as dependent variables. Supplementary Fig. 3 shows the confusion matrix for the classification results, and Table 3 presents comparison of the models used in each task. The results indicate an increase in the classification accuracy for all tasks when oxy-Hb t-values were included alongside task scores. In particular, the accuracy in the gas stove-operation task increased from 44.4% using only task scores to 83.3% when cortical activation was incorporated.

**Table 3:** Comparison of the model performance of the FMA-UE severity classification

Dependent variables	Sensitivity	Specificity	Positive predictive value	Negative predictive value	Overall accuracy
**Gas stove operation**					
Task score	62.5%	30.0%	41.7%	50.0%	44.4%
Task score + oxy-Hb t-value in M1	87.5%	80.0%	77.8%	88.9%	83.3%
**Watering the garden**					
Task score	75.0%	70.0%	66.7%	77.8%	72.2%
Task score + oxy-Hb t-value in M1	87.5%	80.0%	77.8%	88.9%	83.3%
**Hitting the target**					
Task score	37.5%	50.0%	37.5%	50.0%	44.4%
Task score + oxy-Hb t-value in M1	37.5%	70.0%	50.0%	58.3%	55.6%
**Turning off the lights**					
Task score	75.0%	70.0%	66.7%	77.8%	72.2%
Task score + oxy-Hb t-value in M1	87.5%	80.0%	77.8%	88.9%	83.3%

M1, primary motor cortex; oxy-Hb, oxyhemoglobin

## Discussion

This study highlights the utility of fNIRS for monitoring cortical activation in patients with stroke undergoing digital rehabilitation, demonstrating that cortical activation patterns are correlated with motor performance. Our results indicate that distinct patterns of cortical activation are correlated with different levels of motor performance. Notably, significant activations in ipsilesional M1, S1, and contralateral PFC were associated with different motor functions, as indicated by the FMA-UE scores. Our findings indicate that ipsilesional M1 and motor performance were positively correlated whereas S1 and other regions were negatively correlated. The use of the K-NN algorithm, incorporating task performance scores with fNIRS data such as oxy-Hb t-values from the ipsilesional M1, improved the classification accuracy of functional status from 44.4 to 83.3% for specific digital rehabilitation tasks.

This study elucidated the effect of digital rehabilitation on cortical activation in patients with stroke, as measured through fNIRS, highlighting increased activity in key brain areas such as the ipsilesional M1, S1, and contralateral PFC when executing tasks versus the resting state. The activation of these areas, particularly the ipsilesional hemisphere, is essential for motor recovery and neurostimulation [31], highlighting the importance of targeting the ipsilesional M1 and addressing the role of S1 following a stroke [32, 33]. Evidence suggests that both motor and somatosensory areas are important for rehabilitation, with interventions such as digital systems and VR-based rehabilitation promoting functional recovery and neural plasticity by stimulating the sensorimotor cortex [34, 35]. This study supports the efficacy of digital rehabilitation in activating not only motor regions but also areas important for motor planning, reinforcing the therapeutic potential of such programs in stroke rehabilitation.

This study also demonstrated the compensatory role of contralesional PFC in motor rehabilitation, which substitutes or strengthens the functions of stroke-affected regions [36, 37]. The ability of contralesional PFC to form new neural pathways and assume roles for damaged areas highlights a key adaptation mechanism in poststroke recovery [38, 39]. Activation of contralesional PFC supports the reorganization of neural networks during rehabilitation, highlighting its importance in functional recovery, particularly for the rehabilitation of the paralyzed upper limb. This finding significantly contributes to our understanding of the compensatory potential of the contralesional hemisphere in stroke recovery.

This study identified various brain activation patterns corresponding to the severity of motor impairment, as indicated by the FMA-UE scores, demonstrating the differential engagement of brain regions during rehabilitation tasks. Unlike those in healthy individuals, the movements of paralyzed hands in patients with stroke stimulate increased neural activity across the hemispheres, with marked hyperactivity in the SMA and intact motor cortical areas near lesions [40]. This hyperactivity, particularly in the SMA and PPC among patients with severe motor impairments, indicates a compensatory response to upper limb dysfunction [41]. In light of the observed hyperactivity in the PPC among patients with severe motor impairments, the role of the parietal lobe as a critical hub for executive functions and its association with executive dysfunction and cardiovascular risk factors in patients with stroke becomes particularly relevant [42, 43]. This hyperactivity may not only reflect a compensatory mechanism but also emerge as a promising therapeutic indicator, particularly within interventions such as mirror therapy where activity in the precuneus signals treatment effectiveness [44]. These observations indicated the significance of targeting brain regions with high resting metabolic rates, such as the PPC, to establish efficient rehabilitation strategies [45]. Notably, our study observed no significant task-induced brain activation areas during the digital rehabilitation tasks among the group with “notable to full upper limb function.” This absence of significant activation suggested that patients with high functionality, who have already achieved substantial recovery, no longer require intense brain activation. Instead, they use more efficient neural circuits, indicating that successful brain recovery and reorganization have enabled them to perform normal motor functions with less overall brain activation [33, 46]. This is consistent with previous functional MRI findings in patients with chronic stroke, which indicated that greater lesion is correlated with increased cortical hyperactivity [40, 47].

Furthermore, our findings demonstrate a correlation between cortical activation across various brain regions and upper limb functional levels, indicated by a significant positive correlation between ipsilesional M1 and upper limb function. Conversely, the S1, frontal, and parietal lobes showed significant negative correlations with the motor function scores. These results indicate that patients with superior upper limb function rely more on the primary motor cortex whereas those with more severe impairment use compensatory cortical areas, highlighting the adaptive capacity of the brain in response to injury and rehabilitation [47]. In considering the implications of negative correlations in cortical activation, it ws imperative to reflect on findings from previous research. Baskett et al. (1996) noted sensorimotor impairments on the unaffected side in patients with right-lesion strokes, indicating compensatory adaptations associated with S1–M1 coupling in the affected hemisphere—observations that are consistent with our own findings [48]. In addition, Ying et al. (2023) observed a negative correlation between motor function and the resting-state functional connectivity strength of the left S1 and M1 [49], further supporting the notion that improved motor function corresponds with reduced dependence on these brain areas. These examples highlighted the complex neural adaptations during stroke recovery, where decreased activation in specific regions indicated effective adaptation and rehabilitation.

The incorporation of fNIRS-derived cerebral hemodynamic data with digital rehabilitation task performance scores significantly enhanced the accuracy of the classification of rehabilitation levels. The combination of machine learning techniques and fNIRS signals represents a noteworthy advancement in neurorehabilitation, providing a more nuanced understanding of a patient’s neural status and the customization of interventions. The use of fNIRS in brain monitoring has attracted attention in recent research [50–52], with studies achieving > 85% accuracy in differentiating between stroke types and healthy individuals using fNIRS data combined with machine learning algorithms [53]. Moreover, the use of random forest algorithms to analyze fNIRS signals during motor tasks has shown promise in distinguishing patients with major depressive disorder, demonstrating an accuracy of 91.13% [54]. These findings highlight the transformative role of fNIRS in rehabilitation strategies, paving the way for personalized, brain-centric rehabilitation methods. This study contributes to the growing field of stroke rehabilitation research by highlighting the importance of further exploration and optimization of cerebral hemodynamic data integration to improve patient outcomes.

Our findings emphasized the significant potential for tailoring rehabilitation programs to the unique needs of stroke survivors. By examining diverse task performances and the distinct patterns of cortical activation, our study provided valuable insights for the selection of rehabilitation tasks that target varying aspects of motor performance. This approach aligned with the evolving paradigm of personalized rehabilitation, emphasizing interventions tailored to each patient’s specific neural and functional profile. The use of the K-NN algorithm to classify the severity of motor impairment by analyzing task performance scores and cortical activation data illustrated the contribution of our research to more effective, customized rehabilitation strategies. The identification of tasks that elicited significant activation in key areas for motor recovery allows for their strategic integration into rehabilitation programs, thereby enhancing effectiveness. Transitioning these findings into clinical practice necessitates further validation in larger, more diverse patient cohorts. Future research should focus on the development of comprehensive guidelines for implementing task-specific rehabilitation interventions, facilitating the customization of therapy for optimal recovery outcomes.

This study provides important insights but also has limitations that future research should address. The small sample size limits the generalizability of the findings; thus, large-sample studies are warranted to better understand cortical activation patterns in patients with stroke during digital rehabilitation. The cross-sectional design, involving only a single measurement session, restricts our view of the long-term effects of digital rehabilitation on recovery and neural plasticity. Longitudinal studies are necessary to understand these effects over time. Furthermore, focusing on patients at specific rehabilitation stages and with certain stroke characteristics may narrow the applicability of the results. Future studies should involve a wider range of stroke types and recovery stages to comprehensively evaluate the effect of digital rehabilitation across diverse patient groups. Although the use of machine learning algorithms to classify rehabilitation levels is innovative, such algorithms require further refinement and validation to enhance their accuracy and adaptability to the dynamic nature of stroke recovery. Overall, this study highlights the potential of integrating cerebral hemodynamic data in stroke rehabilitation, calling for more comprehensive, varied, and technologically advanced research to optimize the rehabilitation process for stroke survivors.

## Conclusions

This study demonstrates the significant role of fNIRS in the monitoring of cortical activation during digital rehabilitation programs for patients with stroke. Notable activations in the ipsilesional M1, S1, and contralateral PFC were found during digital rehabilitation tasks. These activations varied with motor impairment severity, reflecting the plasticity of the brain and its adaptive response to rehabilitation. Notably, the integration of a K-NN machine learning algorithm with task performance and fNIRS data significantly improved the classification of the participants’ motor impairment severity. The results also emphasize the value of fNIRS as a noninvasive, cost-effective neuroimaging tool in digital stroke rehabilitation. These results may support a multidimensional approach that combines behavioral and cerebral hemodynamic data to customize rehabilitation interventions and set a foundation for future research to optimize stroke rehabilitation outcomes using this technique.

### Electronic supplementary material

Below is the link to the electronic supplementary material.

Supplementary Material 1

## Data Availability

The raw data supporting the conclusions of this article will be made available by the authors upon request.
