# Supplementary material for: Use of cortical hemodynamic responses in digital therapeutics for upper limb rehabilitation in patients with stroke
Source: J Neuroeng Rehabil. 2024 Jul 10;21:115. doi: 10.1186/s12984-024-01404-y (PMC11238451; doi:10.1186/s12984-024-01404-y)
Supplement: Supplementary file 1 — Supplementary Material 1 [file 12984_2024_1404_MOESM1_ESM.docx]

**Supplementary Figure 1. CONSORT flow chart**


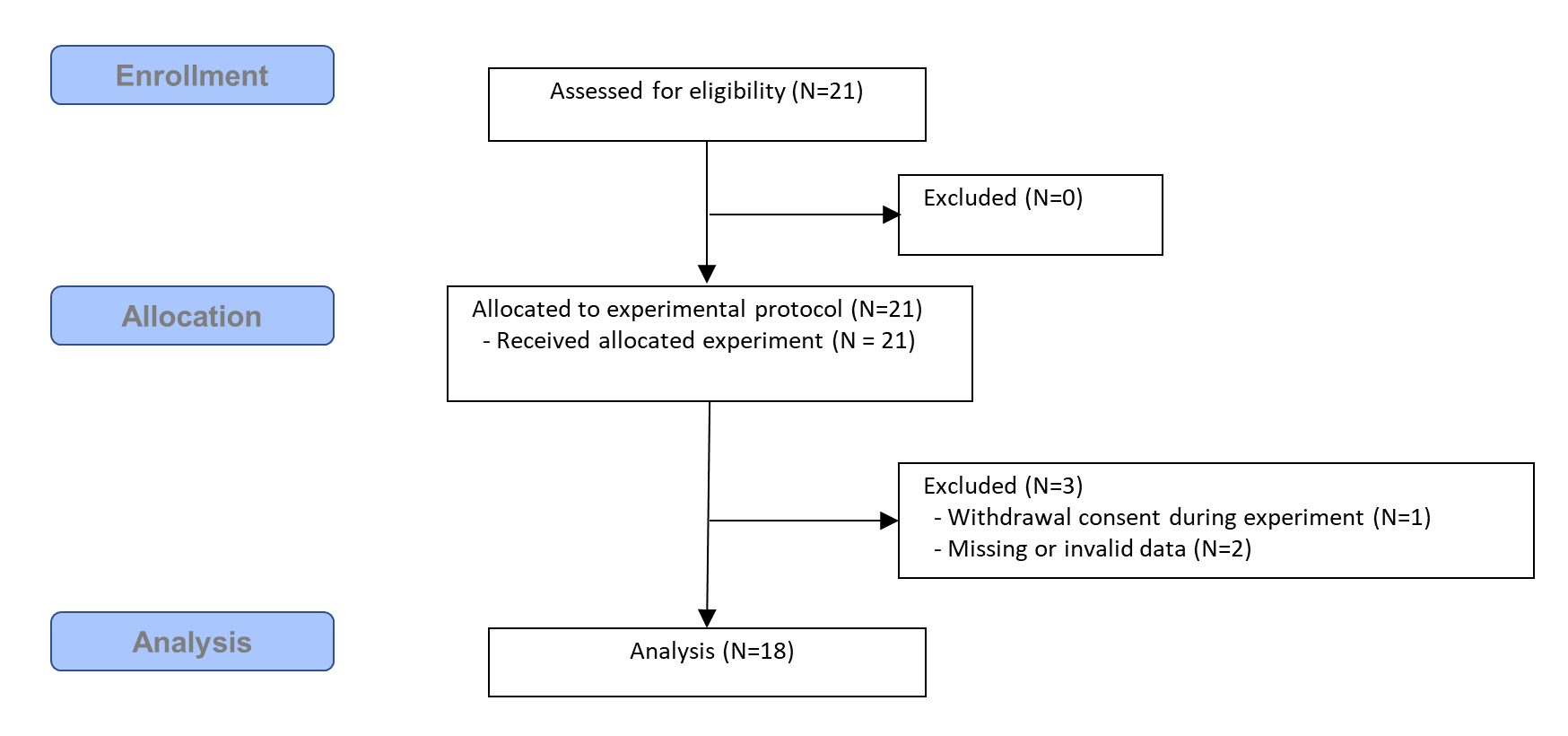


**Supplementary Figure 2. Correlation map between upper limb motor function, task performance, and cortical activation. Red indicates a positive correlation, and blue represents a negative correlation; the deeper the color, the higher the correlation. *P*-values < 0.05 are in bold. FMA-UE, Fugl–Meyer assessment for upper extremity.**

**
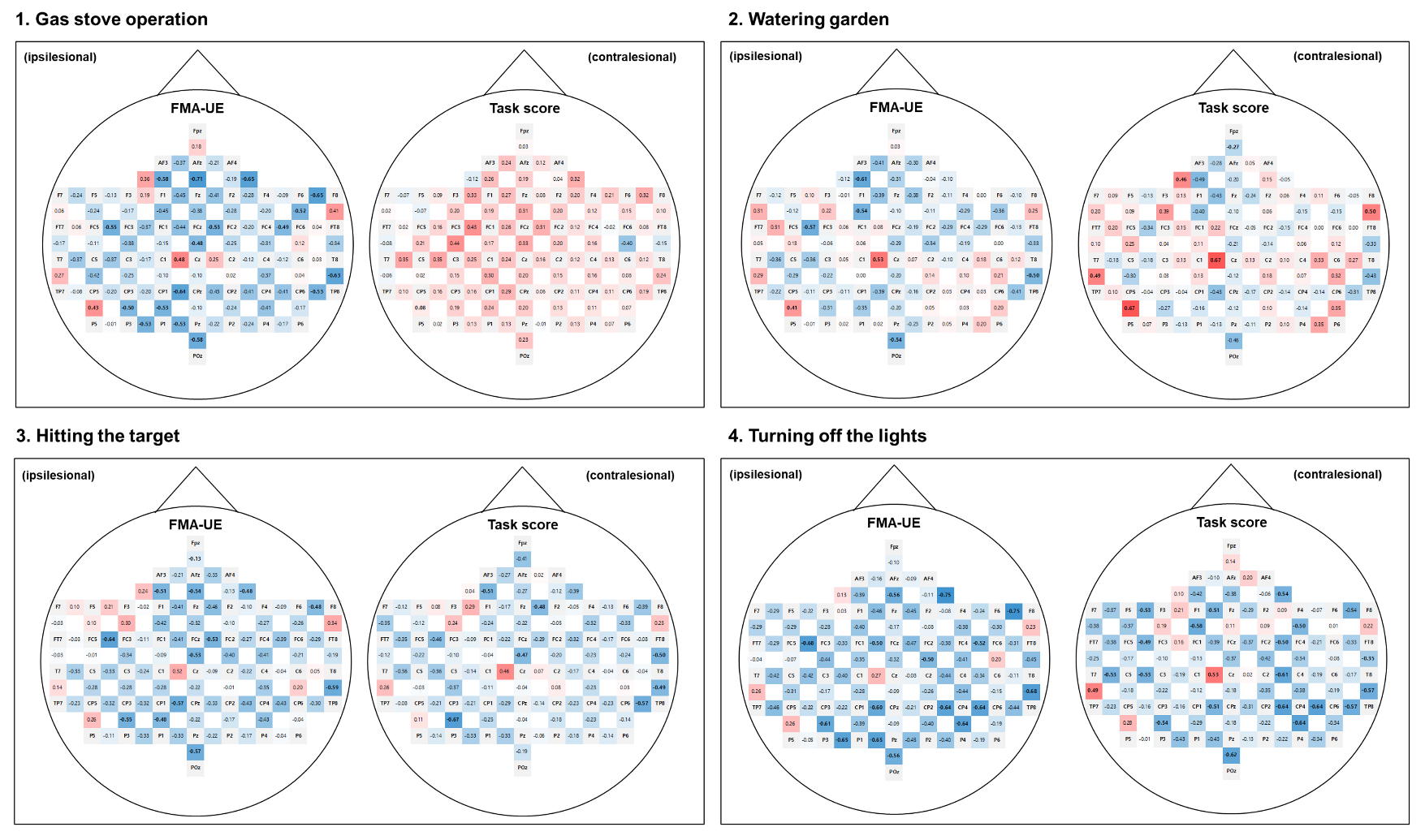
**

**Supplementary Figure 3. Confusion matrix of the K-NN classification**

**
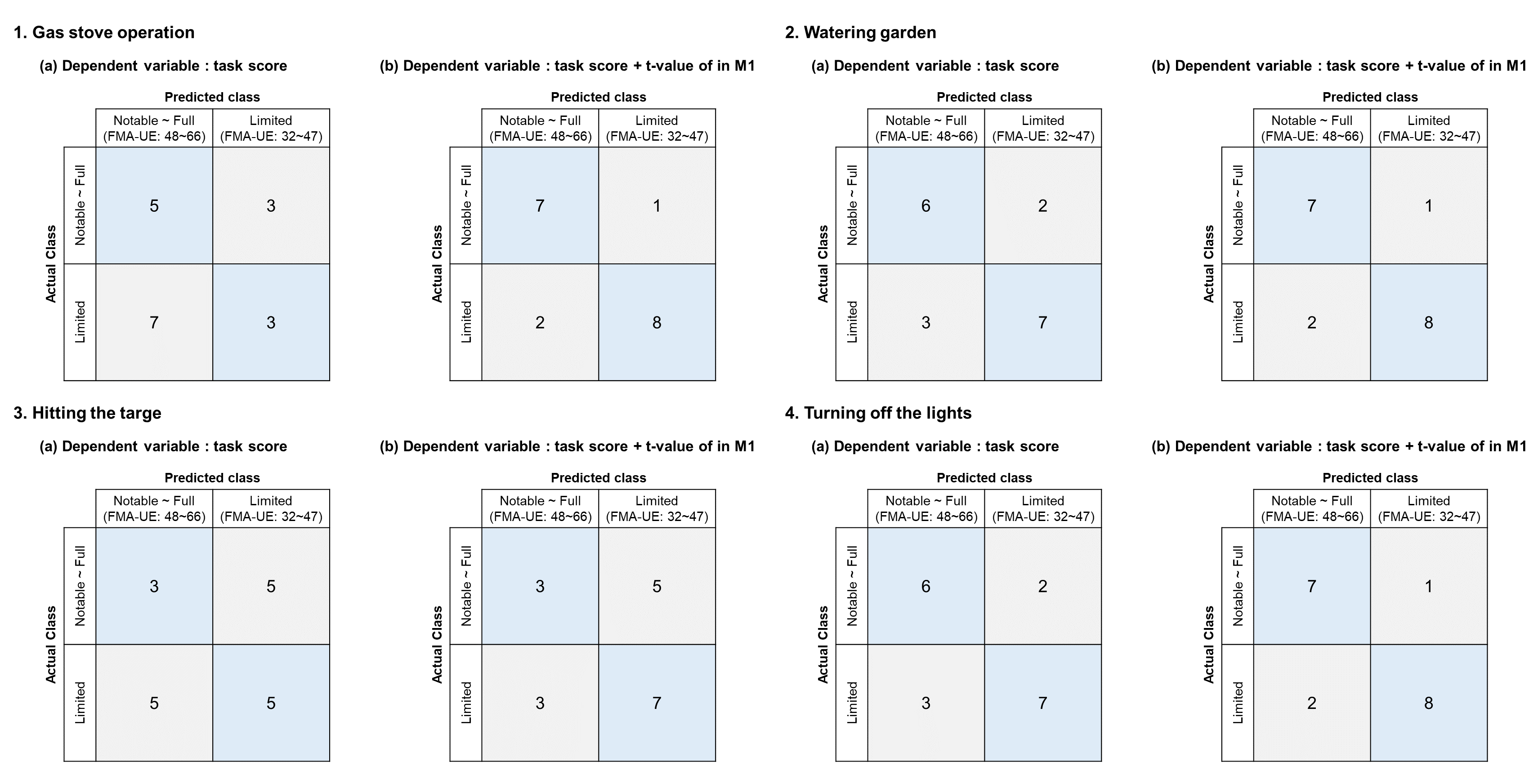
**

**Supplementary Table 1. Position information of functional near-infrared spectroscopy (fNIRS) channels**

| **Ch** | **Source-detector**  **(10/20 system)** | **MNI coordinator**  **(x, y, z)** | | | **Brodmann area**  **(major landmark)** |
| --- | --- | --- | --- | --- | --- |
| 1 | CP1-CP3 | −39 | −48 | 60 | Somatosensory association cortex |
| 2 | CP1-C1 | −27 | −36 | 71 | Primary motor cortex |
| 3 | CP1-CPz | -16 | −50 | 72 | Primary somatosensory cortex |
| 4 | CP1-P1 | −24 | −62 | 62 | Somatosensory association cortex |
| 5 | C3-CP3 | −52 | −34 | 52 | Primary somatosensory cortex |
| 6 | C3-C1 | −42 | −20 | 62 | Primary motor cortex |
| 7 | C3-FC3 | −50 | −3 | 50 | Pre-motor and supplementary motor cortex |
| 8 | C3-C5 | −60 | -18 | 37 | Primary motor cortex |
| 9 | Cz-C1 | -17 | −20 | 74 | Primary motor cortex |
| 10 | Cz-FCz | -1 | −4 | 72 | Pre-motor and supplementary motor cortex |
| 11 | Cz-C2 | 17 | −21 | 75 | Primary motor cortex |
| 12 | Cz-CPz | 1 | −35 | 75 | Primary motor cortex |
| 13 | FC1-C1 | −26 | −5 | 68 | Pre-motor and supplementary motor cortex |
| 14 | FC1-FC3 | −38 | 12 | 55 | Pre-motor and supplementary motor cortex |
| 15 | FC1-FCz | -13 | 12 | 67 | Pre-motor and supplementary motor cortex |
| 16 | FC1-F1 | −23 | 26 | 56 | Includes frontal eye fields |
| 17 | Fz-FCz | 1 | 27 | 58 | Includes frontal eye fields |
| 18 | Fz-F2 | 10 | 41 | 50 | Dorsolateral prefrontal cortex |
| 19 | Fz-AFz | 2 | 50 | 39 | Dorsolateral prefrontal cortex |
| 20 | Fz-F1 | −9 | 41 | 50 | Dorsolateral prefrontal cortex |
| 21 | FC2-FCz | 14 | 13 | 66 | Pre-motor and supplementary motor cortex |
| 22 | FC2-F2 | 24 | 26 | 55 | Includes frontal eye fields |
| 23 | FC2-FC4 | 39 | 12 | 54 | Pre-motor and supplementary motor cortex |
| 24 | FC2-C2 | 27 | −4 | 68 | Pre-motor and supplementary motor cortex |
| 25 | C4-CP4 | 53 | −35 | 52 | Primary somatosensory cortex |
| 26 | C4-C2 | 42 | −21 | 62 | Primary motor cortex |
| 27 | C4-FC4 | 52 | −4 | 48 | Pre-motor and supplementary motor cortex |
| 28 | C4-C6 | 62 | −20 | 37 | Primary motor cortex |
| 29 | CP2-CP4 | 39 | −49 | 60 | Somatosensory association cortex |
| 30 | CP2-C2 | 28 | −36 | 71 | Primary motor cortex |
| 31 | CP2-CPz | 17 | −50 | 73 | Primary somatosensory cortex |
| 32 | CP2-P2 | 25 | −62 | 63 | Somatosensory association cortex |
| 33 | F3-FC3 | −45 | 25 | 41 | Dorsolateral prefrontal cortex |
| 34 | F3-F1 | −31 | 39 | 41 | Dorsolateral prefrontal cortex |
| 35 | F3-F5 | −46 | 39 | 26 | Pars opercularis, part of Broca’s area |
| 36 | FC5-FC3 | −55 | 12 | 34 | Pars opercularis, part of Broca’s area |
| 37 | FC5-C5 | −62 | −3 | 23 | Subcentral area |
| 38 | FC5-FT7 | −59 | 11 | 9 | Retrosubicular area |
| 39 | FC5-F5 | −56 | 24 | 20 | Pars triangularis Broca’s area |
| 40 | F7-FT7 | −54 | 21 | −4 | Temporopolar area |
| 41 | F7-F5 | −53 | 37 | 6 | Pars opercularis, part of Broca’s area |
| 42 | T7-C5 | −65 | -18 | 4 | Superior temporal gyrus |
| 43 | T7-TP7 | −63 | 9 | -12 | Superior temporal gyrus |
| 44 | T7-FT7 | −63 | −9 | -12 | Middle temporal gyrus |
| 45 | P3-CP3 | −46 | −61 | 46 | Somatosensory association cortex |
| 46 | P3-P1 | −32 | −73 | 47 | Somatosensory association cortex |
| 47 | P3-P5 | −46 | −72 | 30 | Angular gyrus, part of Wernicke’s area |
| 48 | Pz-CPz | 2 | −61 | 66 | Somatosensory association cortex |
| 49 | Pz-P1 | -13 | −73 | 56 | Somatosensory association cortex |
| 50 | Pz-P2 | 15 | −73 | 57 | Somatosensory association cortex |
| 51 | Pz-POz | 2 | −80 | 66 | Somatosensory association cortex |
| 52 | P4-CP4 | 46 | −62 | 47 | Somatosensory association cortex |
| 53 | P4-P2 | 33 | −74 | 48 | Somatosensory association cortex |
| 54 | P4-P6 | 47 | −72 | 30 | Angular gyrus, part of Wernicke’s area |
| 55 | CP5-CP3 | −63 | −32 | 23 | Superior temporal gyrus |
| 56 | CP5-C5 | −63 | −32 | 23 | Retrosubicular area |
| 57 | CP5-TP7 | −65 | −44 | 5 | Superior temporal gyrus |
| 58 | CP5-P5 | −57 | −57 | 21 | Angular gyrus, part of Wernicke’s area |
| 59 | CP6-CP4 | 58 | −48 | 38 | Superior temporal gyrus |
| 60 | CP6-C6 | 65 | −33 | 23 | Superior temporal gyrus |
| 61 | CP6-TP8 | 65 | −44 | 6 | Superior temporal gyrus |
| 62 | CP6-P6 | 58 | −58 | 22 | Angular gyrus, part of Wernicke’s area |
| 63 | T8-C6 | 67 | -19 | 4 | Superior temporal gyrus |
| 64 | T8-TP8 | 66 | 8 | -12 | Superior temporal gyrus |
| 65 | T8-FT8 | 66 | −8 | -12 | Middle temporal gyrus |
| 66 | FC6-F6 | 58 | 24 | 18 | Pars triangularis Broca’s area |
| 67 | FC6-FC4 | 56 | 12 | 33 | Pars opercularis, part of Broca’s area |
| 68 | FC6-C6 | 64 | −5 | 22 | Subcentral area |
| 69 | FC6-TP8 | 61 | 11 | 8 | Retrosubicular area |
| 70 | F8-F6 | 55 | 36 | 5 | Pars opercularis, part of Broca’s area |
| 71 | F8-FT8 | 57 | 21 | −4 | Temporopolar area |
| 72 | F4-F6 | 46 | 38 | 24 | Pars opercularis, part of Broca’s area |
| 73 | F4-FC4 | 44 | 25 | 40 | Dorsolateral prefrontal cortex |
| 74 | F4-F2 | 30 | 40 | 41 | Dorsolateral prefrontal cortex |
| 75 | AF4-F6 | 40 | 50 | 16 | Dorsolateral prefrontal cortex |
| 76 | AF4-F2 | 22 | 52 | 33 | Dorsolateral prefrontal cortex |
| 77 | AF4-AFz | 13 | 61 | 24 | Frontopolar area |
| 78 | AF3-AFz | -12 | 62 | 23 | Frontopolar area |
| 79 | AF3-F1 | −23 | 52 | 32 | Dorsolateral prefrontal cortex |
| 80 | AF3-F5 | −39 | 50 | 17 | Dorsolateral prefrontal cortex |
| 81 | Fpz-AFz | 1 | 64 | 14 | Frontopolar area |

**Supplementary Table 2. Performance results of each task in the digital rehabilitation program**

| **Task (movement)** | | **Value** |
| --- | --- | --- |
| Gas stove operation | Maximum level | 5.0 ± 0.0 (5, 5) |
| (Supination and pronation) | Average score | 99.9 ± 0.2 (99, 100) |
|  | Average score < 70 points (N, %) | 0 (0%) |
| Watering the garden | Maximum level | 3.8 ± 1.4 (1, 5) |
| (grasping) | Average score | 78.6 ± 27.1 (0, 100) |
|  | Average score < 70 points (N, %) | 6 (33.3%) |
| Hitting the target | Maximum level | 1.2 ± 0.5 (1, 3) |
| (pressing the button) | Average score | 34.0 ± 20.9 (0, 71.2) |
|  | Average score < 70 points (N, %) | 17 (94.4%) |
| Turning off the lights | Maximum level | 4.5 ± 1.2 (1, 5) |
| (reaching and touching) | Average score | 85.9 ± 18.1 (32.14, 100) |
|  | Average score < 70 points (N, %) | 3 (16.7%) |

Values are expressed as mean ± SD (min, max). SD, standard deviation; N, number
